# Can we forecast poor outcome in herpes simplex and varicella zoster encephalitis? A narrative review

**DOI:** 10.3389/fneur.2023.1130090

**Published:** 2023-06-26

**Authors:** Lena S. Abbuehl, Eveline Hofmann, Arsany Hakim, Anelia Dietmann

**Affiliations:** ^1^Department of Neurology, Inselspital, Bern University Hospital, University of Bern, Bern, Switzerland; ^2^Department of Infectious Diseases, Inselspital, Bern University Hospital, University of Bern, Bern, Switzerland; ^3^Institute of Diagnostic and Interventional Neuroradiology, Inselspital, Bern University Hospital, University of Bern, Bern, Switzerland

**Keywords:** viral encephalitis, meningoencephalitis, prognosis, outcome, varicella zoster encephalitis, herpes zoster encephalitis

## Abstract

Herpes simplex virus (HSV) and varicella zoster virus (VZV) are among the most commonly diagnosed infectious causes of sporadic encephalitis worldwide. Despite treatment, mortality and morbidity rates remain high, especially for HSV encephalitis. This review is intended to provide an overview of the existing scientific literature on this topic from the perspective of a clinician who is confronted with serious decisions about continuation or withdrawal of therapeutic interventions. We performed a literature review searching two databases and included 55 studies in the review. These studies documented or investigated specifically outcome and predictive parameters of outcome of HSV and/or VZV encephalitis. Two reviewers independently screened and reviewed full-text articles meeting the inclusion criteria. Key data were extracted and presented as a narrative summary. Both, HSV and VZV encephalitis have mortality rates between 5 and 20% and complete recovery rates range from 14 to 43% for HSV and 33 to 49% for VZV encephalitis. Prognostic factors for both VZV and HSV encephalitis are older age and comorbidity, as well as severity of disease and extent of magnetic resonance imaging (MRI) lesions on admission, and delay in treatment initiation for HSV encephalitis. Although numerous studies are available, the main limiting factors are the inconsistent patient selection and case definitions as well as the non-standardised outcome measures, which hampers the comparability of the studies. Therefore, larger and standardised observational studies applying validated case definitions and outcome measures including quality of life assessment are required to provide solid evidence to answer the research question.

## 1. Introduction

Encephalitis is an inflammatory disease of the central nervous system (CNS), sometimes associated with meningitis, neuritis, radiculitis and/or myelitis (1). The estimated annual incidence of all types of encephalitis worldwide is between 1 and 14 cases per 100,000 (2–5). Clinically, encephalitis is defined as altered mental status lasting for ≥24 h, accompanied by evidence of brain parenchymal inflammation. This includes fever, new-onset seizures, focal neurological signs, cerebrospinal fluid (CSF) pleocytosis and/or abnormal findings in magnetic resonance imaging (MRI) or on electroencephalography (EEG) (1). Viral encephalitis is a serious condition with overall mortality rates of up to 30% depending on the causal agent in non-tropical regions (6–8). Up to 75% of long-term survivors of infectious encephalitis have persisting signs and symptoms that significantly impair their quality of life, including cognitive deficits, behavioural and speech disorders, epileptic seizures, frequent headaches and fatigue (7, 9, 10). The socioeconomic impact of infectious encephalitis is considerable: one quarter to half of the patients who were previously employed are unable to return to work (7, 9, 11).

Herpes simplex virus (HSV) encephalitis is the most commonly diagnosed viral encephalitis in industrialised nations (3, 6, 11–14). Besides being one of the most frequent causes, HSV infections of the CNS are also among the most severe of all viral infections of the human brain (14). Typically, after a prodromal phase, patients present with non-specific signs and symptoms such as seizures, abnormal behaviour, impaired consciousness and focal neurological deficits (15). Untreated HSV encephalitis has very high mortality rates of up to 70 and 97% of survivors do not regain their previous level of function (14). The introduction of aciclovir treatment has significantly improved outcome following HSV encephalitis (16, 17).

Another important and treatable herpes virus causing encephalitis is the varicella zoster virus (VZV) (1, 6, 11). Chickenpox is the primary form of VZV infection, occurring mainly in children, and herpes zoster due to reactivation of the virus occurs mostly in adults (3). Less common manifestations of VZV reactivation, and rarely of primary infection, affect the CNS and peripheral nervous system (PNS). They include encephalitis, meningitis, cerebellitis, myelitis and vasculopathy/stroke as well as radiculopathy, peripheral facial palsy and Ramsay Hunt syndrome (18).

In the emergency ward, as soon as viral encephalitis is suspected, the question of outcome and prognosis arises. Knowledge of estimated outcome and prognostic markers is important to optimise case-specific treatment, clinical care and patient information. Many studies, most of them retrospective, have investigated clinical presentation, course of disease including mortality rates and the clinical outcome in survivors. A wide variety of factors – from presenting clinical signs and symptoms, age, comorbidities, interval between onset and hospital admission or initiation of treatment, laboratory parameters and imaging features – have been studied to assess their value as prognostic factors.

As a consultant neurologist working in an intensive care unit (ICU), one must not only be able to inform patients and relatives about the prognosis and expected long-term consequences, but one is also confronted with serious decisions about the continuation or withdrawal of life-sustaining therapies depending on the clinical severity of CNS infections. Against this background, this review is intended to summarise the existing literature and provide an overview of the scientific basis that can be used to assist in making these momentous decisions.

## 2. Methods

We searched MEDLINE/PubMed (National Library of Medicine) for relevant literature and the Cochrane Library for randomised controlled trials on viral encephalitis caused by HSV or VZV describing clinical outcome or prognostic factors published from 1996 to December 2022. Only reports of research in humans were included. The search terms, selection and exclusion of literature are listed in Supplementary Figure S1. Furthermore, we searched the reference lists of the publications included to identify additional studies not detected in the initial search.

### 2.1. Eligibility criteria

Studies were included if they were case-series including more than 10 patients, case–control studies, cohort studies or randomized-controlled trials. We included publications that reported on cases with features of encephalitis or meningoencephalitis that were suspected or confirmed to have been caused by HSV or VZV. Diagnosis had to be confirmed by detection of HSV or VZV in the CSF by polymerase chain reaction (PCR) (5, 19). Patients without PCR or serological microbiological confirmation had to be distinguishable in the final analysis. We excluded studies focusing on other causes of infectious encephalitis or meningoencephalitis, autoimmune mediated encephalitis or non-CNS syndromes, such as isolated myelitis or radiculitis. Inclusion criteria were publication in German or English language and the availability of the full text. Studies performed solely in children were also excluded as childhood cases are likely to represent clinical entities that are distinct from adult cases.

### 2.2. Data extraction

A detailed review of each study was conducted by two independent researchers (LA and AD), during which the following details were extracted: number of patients, cause of encephalitis or meningoencephalitis, clinical syndrome, age, abnormal investigation findings including MRI, outcome measures, factors tested for correlation with outcome and study results. We had a particular interest in publications in which MRI findings were used as markers for prognosis. The CSF parameters we considered applicable were those identified by routine testing, such as protein, glucose, white cell count, or differential cell counts. We recorded MRI abnormalities likely due to encephalitis or meningoencephalitis, or any MRI abnormality if these details were not specified.

## 3. Results

### 3.1. Herpes simplex encephalitis

The studies reviewed are summarised in Table 1 and included 32 retrospective and 8 prospective studies from Europe (France, Denmark, Sweden, Spain, Turkey, Czech Republic, Austria), the United States, Israel, Asia (India, Republic of Korea, Japan, China) and New Zealand. Two multinational studies included data from Arabic countries (Egypt, Iraq, Lebanon) (37, 38). In most studies, the proportion of HSV1 to HSV2 infections was evident. The outcome was generally assessed at discharge and during follow-up periods of 3 to 6 months and/or after 1 year. Eight studies had further follow-up periods of up to 2 years (7, 11, 27, 34, 35, 43, 50) or even up to 11 years (20, 21, 50).

**Table 1:** Reports with data on outcome and/or prognostic factors for HSV or VZV encephalitis.

Reference	Study design	Number of patients	Patient age in years^†^	Time until follow-up	Definition of poor/unfavourable/adverse outcome or measures of outcome	Outcome	Prognostic factors/main findings
HSV							
McGrath et al. (20) and Utley et al. (21)	Retrospective, monocentre, New Zealand	*n* = 42 (20) *n* = 20 (21)	Median 47 (3 months – 91 years)	Mortality 6 months, clinical sequelae after 6 months – 11 years	GOS	14% mortality at 6 months 19% GOS 2–3 21% GOS 4 48% GOS 5 (only 1/20 asymptomatic and normal neurological examination)	*t*-Test: Older age, stupor or coma before acyclovir start, delayed treatment start, abnormal initial CT-scan with worse outcome
Raschilas et al. (22)	Retrospective, multicentre, France	Encephalitis *n* = 93 (HSV2 *n* = 1)	16–88	6 months	GOS 1–2*	15% mortality 65% favourable (GOS 3–5, 14% complete recovery) 35% poor	Univariate analysis: Low GCS and higher APACHE score on admission Multivariate analysis Delay in aciclovir therapy >2d after admission and SAPS II score ≥ 27 at admission
Kamei et al. (23)	Retrospective, monocentre, Japan	Encephalitis *n* = 18	19–74	3 months	Moderate or severe sequelae: motor, speech, memory or seizure disorder, supportive care or death	17% mortality 61% good (39% complete recovery) 39% poor	Non-parametric test of hypothesis CSF viral load not associated with poor outcome
Kamei et al. (24), Kamei et al. (25) and Taira et al. (26)	Retrospective, non-randomised comparative study, monocentre, Japan	Encephalitis *n* = 45 (24) *n* = 20 (25) *n* = 23 (26)	17–77	3 months	Moderate or severe sequelae: motor, speech, memory or seizure disorder, supportive care or death (24) Prolonged course (no neurological improvement after 14 days of aciclovir treatment) (26)	11% mortality 58% good (31% complete recovery) 42% poor Prolongedcourse n = 8 (3 death, 1 severe sequelae)	Single and multiple logistic regression analysis (24): Older age, low GCS at initiation of aciclovir, no administration of corticosteroids Mann–Whitney U test (25, 26) Higher initial CSF IFNɣ and maximum CSF IL6 associated with poor outcome Lesions on initial CT associated with prolonged course and prolonged course associated with poor outcome
Hjalmarsson et al. (27)	Retrospective, nationwide, Sweden	Encephalitis *n* = 48	Median 58.5 (IQR 47–66)	30 months, not clearly described	Poor: needs continuous care, at home or in an institution, or death	29% mortality 44% good (mild to moderate outcome) 56% poor	Cox regression model: CSF viral load and IgG levels not associated
Riera-Mestre et al. (28)	Retrospective, monocentre, Spain	Encephalitis *n* = 35	Median 53 (IQR 37–71)	At discharge and at 6 months	mRS ≥3	8.6% mortality 71% good 29% poor	Uni- and multivariate analyses: Age, serum albumin on admission, duration of fever after initiation of treatment
Stahl et al. (29)	Prospective multicentre, France	Encephalitis *n* = 55 (*n* = 3 HSV2, *n* = 10 not determined)	1 month – 89 years	<3 months after discharge	Death, discharge to long-term facility, major impairment or Rankin scale score ≥ 3	5% mortality 53% favourable 47% poor	Non-parametric test of hypothesis and uni- and multivariate analysis: Dosage, duration or time between onset and treatment not associated with poor outcome
Poissy et al. (8)	Retrospective, monocentre, France	Encephalitis *n* = 43	Median 61 (IQR 50–69)	6 months	GOS 1–2*	33% mortality 55% good 45% poor (GOS 4–5)	Univariate analysis: Higher APACHE score, delay in treatment, older age, RBC in CSF; CSF viral load not associated
Tan et al. (30)	Retrospective case–control review, United States	Encephalitis *n* = 29 (immunocompetent versus immunocompromised)	26–79	1 months after aciclovir discontinuation	KPSS	Immunocompromised: 26% mortality, lower CSF cell count, significantly lower KPSS; immunocompetent: 7% mortality	Multivariate linear regression model: Lower CSF cell count, delay in treatment initiation, immunosuppressive state
Riancho et al. (31)	Retrospective, monocentre, Spain	Encephalitis *n* = 26 (HSV1 positive *n* = 16, negative or not done *n* = 10)	23–90	At discharge	Poor (death or sequelae) or favourable outcome not clearly defined	11% mortality 32% favourable 68% poor	Fisher test: Older age, fever (>38°), disorientation, abnormal early CT
Sili et al. (32)	Retrospective, multicentre, Turkey	Encephalitis *n* = 106 (PCR positive *n* = 55, 91% HSV1)	18–83	At least 6 months after discharge	Severe sequelae or fatality	8% fatality 73% favourable (23% complete recovery) 27% poor	Univariate analysis: Duration of disease before admission, extent of brain involvement on admission MRI
Jouan et al. (33)	Retrospective monocentre cohort study, France	Encephalitis *n* = 14 (HSV1 *n* = 13)	22–82	1 year	GOS	14% mortality 43% GOS 5 21% GOS 4 21% GOS 3	Spearman’s rank correlation: Initial brain imaging not predictive of risk for brain herniation
Gnann et al. (34) and Westman et al. (35, 36)	Prospective multicentre placebo-controlled randomised trial study, Sweden	Encephalitis *n* = 87 (34) Encephalitis *n* = 53 (35)	14–83	90 days, 6, 12 and 24 months	MDRS	5% Mortality At 12/24 months: 86/90% no or mild impairment (121–144) 14/11% moderate/severe/very severe impairment (<121)	Multivariable linear regression models: Valaciclovir follow-up treatment not beneficial (34) CSF neurofilament, age and presence of CSF anti-NMDAR IgG (36)
Erdem et al. (37) and Cag et al. (38)	Retrospective, multicentre, 10 countries	*n* = 496 (HSV1 *n* = 351, HSV2 *n* = 83, undefined *n* = 62)	Mean 50.6 (±18.3 SD)	Unknown	Death or survivors with sequelae	10% mortality 47% favourable 53% unfavourable	Multivariate model: Age, male sex, low GCS (<5) and time from onset to treatment of >2 days (37) Clinical presentation with encephalitis, length of hospital stay (38)
Kalita et al. (39)	Retrospective, monocentre, India	*n* = 40	Median 25 (range 1–78)	Discharge, 3, 6 and 12 months	mRS 3–5, persistance sequelae	30% mortality Outcome 1 year: 16% poor 32% complete recovery	n.a.
Kim et al. (40)	Retrospective, multicentre, Republic of Korea	Encephalitis *n* = 29 (HSV1 *n* = 22)	3–77	6 months	GOS 3–5	0% mortality 48% favourable (34% complete recovery) 52% poor	Uni- and multivariate analysis: Epileptic seizures and severe EEG abnormality at admission; No association with age, MRI lesions, time to aciclovir treatment
Singh et al. (41)	Retrospective monocentre study, United States	HSV1 *n* = 33 HSV2 *n* = 9	Median 66 (IQR 54–78)	At discharge and after 6–12 months	mRS 3–6	Discharge: HSV1 64%, HSV2 56% 6–12 months: HSV1 31%, HSV2 44%	Uni- and multivariate analysis Older age, coma, restricted diffusion on MRI and aciclovir started after first day of admission No association with seizures, focal deficits, EEG abnormalities or location or extension of FLAIR/T2 abnormalities
Jørgensen et al. (42)	Retrospective population- based nationwide registry cohort study, Denmark	*n* = 230 (HSV1, −2 and – not specified)	Median 61 (IQR 49–72)	30, 60 days and 1 year	Mortality	Mortality 8.3, 11.3 and 18.6%	Uni- and multivariate analyses Older age, presence of comorbidity (Charlson comorbidity index ≥1)
Armangue et al. (43)	Prospective and retrospective observational, multicentre, Spain	Encephalitis prospective *n* = 55 (HSV1 or 2 not specified) retrospective *n* = 48	Median 50 (IQR 5–68); < 4 years *n* = 13, 5–12 years *n* = 5	12 and 24 months	mean mRS	Mean mRS 2 (IQR 1–3) at 6/12 months 27% developed AE within 2 months after HSV encephalitis	*Multivariate logistic regression analysis* Shorter interval (3 weeks) to detection of AE antibodies associated with risk of AE
Bewersdorf et al. (44)	Retrospective, monocentre, Germany	*n* = 18	Mean 54.7 (range 20–90)	n.a.	Mortality, GOS <5	6% mortality 61% poor outcome	
Meyding-Lamadé et al. (45)	Prospective, multicentre treatment study, Germany	*n* = 41	Mean 60.1 ± 13.6	6 and 12 months	mRS 3–6, GOS, EQ-5D, cognitive assessment	6 months: 3.1% mortality, 36.8% mRS >2 11.1% mortality, 44.8% mRS >2	n.a.
Oud (46)	Retrospective, population-based registry, United States	*n* = 1964	21% (18–44 years) 34% (45–64 years) 45% (≥65 years)	n.a.	Mortality, discharge to hospice and rate of ICU admission	8.8% mortality 4.2% discharge to hospice 59.9% ICU admission	Logistic regression: Older Age associated with ICU admission and increased risk of death and discharge to hospice
Jaquet et al. (47), Sarton et al. (48), and de Montmollin et al. (49)	Retrospective, multicentre cohort study, France	Encephalitis *n* = 259 (*n* = 205 HSV1) (47, 49) Encephalitis *n* = 138 (HSV1 *n* = 118) (48) Encephalitis *n* = 273 (49)	54–73 (47, 48), 31–85 (49)	At 90 days after ICU discharge (47, 48) At discharge and after 3 months (49)	mRS 3–6 (47, 48) mRS ≥4 (49)	Jaquet et al. (47) 17% mortality 71% poor (mRS 3–6) Sarton et al. (48) 12% mortality 69% poor Montmollin et al. (49) 14% mortality 37% poor at discharge 24% poor at 3 months	Uni- and multivariate logistic regression model: Body temperature ≥ 38.3°C at admission, need for mechanical ventilation, MRI with >3 brain lobes affected; direct ICU admission protective (47) FLAIR >3 lobes involved, age > 60 years, DWI in left thalamus, SAPS >34 (48) Initial CSF HSV PCR negative (49)
Hansen et al. (50)	Retrospective population- based nationwide registry cohort study, Denmark	CSF HSV1 positive *n* = 208, Comparison cohort *n* = 2080 CSF HSV2 positive *n* = 283, Comparison cohort *n* = 2,830	HSV 1: Median 60 (IQR 41–70), <16 *n* = 16 HSV2: Median 38 (IQR 28–48), <16 *n* = 7	Median 3.7–6.2 years	All-cause mortality, cancer, dementia, epilepsy, health care utilisation, poor social functioning; death or severe disability (receipt of disability pension, nursing home, dementia)	One-year absolute excess mortality rate: 19% HSV1 and 2% HSV2 Mortality rate ratio: 10.9 HSV1 and 8.4 HSV2	Survival analysis and mortality rate ratio and incidence rate ratios Increased risk of death in the first year after HSV CNS infection Increased risk of dementia in the first years after HSV1 (4.6% 5-year risk)
Müller-Jensen et al. (51)	Retrospective, multicentre, Germany	*n* = 25	Median 67 (IQR 56–78)	Median 1 (IQR 0–2) months	Mortality, recovery with sequelae/ongoning symptoms	24% Mortality 56% recovery with sequelae 20% full recovery	n.a.
Mulatero et al. (52)	Retrospective, descriptive, monocentre, France	Encephalitis *n* = 76 (HSV2 *n* = 4)	16–92	Not specified	Need for assistance by another person, disability, or death at discharge Favourable: resumption of professional activity identical to previous activity (full recovery)	12% Mortality 49% favourable (42% complete recovery) 51% poor	Logistic regression analysis: EEG with status epilepticus, persisting confusional state, aphasia or impaired consciousness after 5 days of evolution, >8 days ICU stay, admission-to-MRI delay
HSV and VZV							
Růžek et al. (53)	Retrospective, monocentre, Czech Republic	CNS infection (HSV *n* = 14; VZV *n* = 17)	1–88	No follow-up	Mild or severe course and good outcome (complete recovery) or poor outcome (severe sequelae)	0% Mortality Poor outcome: HSV 50%, VZV 29%	Non-parametric test of hypothesis: No correlation of viral load and outcome
Mailles et al. (7, 11)	Prospective, nationwide cohort study and follow-up study, France	Encephalitis total *n* = 253 VZV *n* = 20 HSV *n* = 55	1 month – 89 years	In hospital and after 3 years	In-hospital mortality (11) GOS 1–4 at follow-up after 3 years (7)	Mortality: VZV 15%, HSV 5% (in hospital) Full recovery: HSV 14%, VZV 33%	Logistic regression: Poor long-term outcome associated with comorbidities, age, level of education and HSV (7)
Kaewpoowat et al. (54)	Retrospective observational, monocentre, United States	Encephalitis (HSV *n* = 20, VZV *n* = 5); meningitis (HSV *n* = 60, VZV = 13)	HSV: 18–82 VZV: 25–88	At discharge, no follow-up	GOS 1–4	Adverse clinical outcome in HSV 12.5% (mortality 0%), and VZV 33% (mortality 6%)	Logistic regression: Comorbidities (Charlson comorbidity score > 1) and encephalitic course
Jordan et al. (55)	Retrospective, monocentre, Germany	Encephalitis (HSV *n* = 15, VZV *n* = 5, EBV *n* = 2)	HSV: Mean 45 (±19 SD) VZV: Mean 65 (±15 SD) EBV: 38 and 67	At discharge	n.a.	Remission: HSV 27%, VZV 20% Mild-to-moderate disability: HSV 73%, VZV 80%	
Lee et al. (56)	Retrospective, monocentre, Republic of Korea	Meningitis and encephalitis (HSV1 *n* = 11, HSV2 *n* = 27, VZV *n* = 42)	16–92	At discharge, no follow-up	In-hospital mortality and neurological sequelae at discharge	Poor HSV1 27%, HSV2 0% and VZV 7%, mortality not specified	Non-parametric test of hypothesis: Encephalitis more common in HSV1 with poor prognosis compared to HSV2 and VZV at discharge
VZV							
Aberle et al. (57)	Retrospective, monocentre, Austria	Encephalitis *n* = 13 Meningitis *n* = 17	11–88	No follow-up	Acute disease severity (encephalitis versus meningitis)	3% mortality, no further data on outcome	Non-parametric test of hypothesis: Higher VZV DNA CSF load in encephalitis versus meningitis
Persson et al. (58)	Retrospective, monocentre, Sweden	Meningitis *n* = 34; Encephalitis *n* = 28 Cranial neuropathies *n* = 20 Encephalopathy *n* = 5 cerebrovascular disease *n* = 6	3 months – 94 years	1, 3 and 6 months	Acute disease severity (encephalitis and meningitis versus other manifestation)	4% mortality 68% with persisting neurological complications at 1 month	Non-parametric test of hypothesis: Higher VZV DNA levels in meningitis/encephalitis versus cranial neuropathies or encephalopathy or stroke
De Broucker et al. (59)	Prospective, monocentre, France (11)	Encephalitis without vasculopathy, *n* = 20	*n* = 3 age 0.5–5 *n* = 17 age 19–86	Discharge and after 3 years	GOS < 5	15% mortality 45% with persistent neurological signs at discharge; 41% GOS 3–4 and 41% with GOS 5 after 3 years	n.a.
Grahn et al. (60, 61)	Prospective, multicentre, case–control study, Sweden	*n* = 24 (61) *n* = 14 (60) Meningitis, encephalitis, radiculitis, neuropathy	19–83	12 months (61) Median 39.5 months (60)	GOS and neurological sequelae (61) Cognitive impairment (60)	All patients with GOS 4 or 5 (encephalitis 71% with sequelae, meningitis 0% with sequelae, neuropathy 80% with sequelae) (61)	*Non-parametric test of hypothesis, Spearman’s rank* CSF NFL, GFAp, S-100β not associated with outcome (61) More frequent cognitive impairment in the domains of speed and attention, executive function, and learning and memory compared to control group (60)
Hong et al. (62)	Retrospective, monocentre, Republic of Korea	Meningitis *n* = 29 Encephalitis *n* = 9	Median 35 (IQR 26–62)	Discharge, 1, 3 and 6 months	In-hospital mortality and neurological sequelae (not further specified)	Mortality 0% Neurological sequelae 7.9, 5.3, 2.6, 2.6%	n.a.
Rottenstreich et al. (63)	Retrospective, Israel	Meningitis *n* = 25; encephalitis *n* = 20	15–82	No follow-up	Acute disease severity	7% mortality (only encephalitis), favourable outcome in all meningitis and other encephalitis cases (not further defined)	Non-parametric test of hypothesis, Kendall’s correlation Higher VZV DNA load in CSF and older age in encephalitis patients
Skripuletz et al. (64)	Retrospective, monocentre, Germany	Any VZV disease *n* = 282; encephalitis *n* = 18, meningitis *n* = 15, myelitis *n* = 1	All age groups, not specified	n.a.	Description of clinical course	Encephalitis: 1/18 died, 7/18 needed rehabilitation (6/7 with severe neuropsychiatric symptoms)	n.a.
Corral et al. (65)	Retrospective, monocentre, Spain	*n* = 98 (cranial neuropathies, encephalitis, radiculopathies, meningitis, vasculitis, myelitis)	Median 66 (IQR 50–78)	At least 6 months	mRS >2	3% mortality 71% complete recovery, 24% mild sequelae (mRS 1)	Binary logistic regression model: Immunosuppression associated with acute severity, but not prognosis Shorter latency between herpes zoster and neurological symptoms associated with unfavourable outcome
Tabaja et al. (66)	Retrospective, monocentre, Lebanon	Meningitis *n* = 16, encephalitis *n* = 4	Mean 49,7 ± 22.2	n.a.	n.a.	No mortality, no neurological sequelae	n.a.
Le Bot et al. (67)	Retrospective, observational, monocentre, France	Meningitis *n* = 21 Meningoencephalitis *n* = 15	Meningitis: Median 38, Encephalitis: Median 72	At discharge, no follow-up	Death or any neurological sequelae at discharge	0% mortality, 33% with neurological sequelae	Exact logistic regression: Age
Herlin et al. (68)	Prospective, nationwide cohort study, Denmark	Encephalitis *n* = 92	Median 75 (IQR 67–83)	At discharge, 1 and 3 months	GOS <5	4, 9 and 11% mortality 69, 55 and 51% unfavourable (at discharge, 1 and 3 months)	Poisson regression: GCS <15, age (>75 years), vasculitis
Omland et al. (69)	Retrospective population-based nationwide registry cohort study, Denmark	VZV cohort *n* = 517 Encephalitis (44%), meningitis (21%), herpes zoster (14%) and other (21%)	Median 59 (IQR 31–77)	1, 2 and 5 years before study inclusion and up to 12 years thereafter		12% mortality in VZV cohort after 1 year and increased risk of dementia and epilepsy	Logistic regression: Immunosuppression and comorbidity (Charlson comorbidity index ≥1) associated with increased risk of VZV DNA detection in CSF
Lenfant et al. (70)	Retrospective, multicentre observational, France	Meningitis *n* = 26 CNS group *n* = 27 PNS group *n* = 16	Meningitis: Median 34 (IQR 24–48), CNS: Median 63 (IQR 52–81), PNS: Median 68 (37–82)	After median 2.9 years	Mortality or incomplete recovery (any persistent symptom or sequelae)	Mortality: only CNS group 36% Unfavourable: 24% meningitis, 82% CNS, 87% PNS group	Multiple logistic regression Older age, prior-to-infection mRS, CNS and PNS affection
Yan et al. (71)	Retrospective, descriptive, monocentre, China	Meningitis *n* = 59 Meningoencephalitis *n* = 15 (only 6/20 patients CSF VZV DNA positive, but herpes zoster as inclusion criterion)	Meningitis: 26–87, Meningoencephalitis: 48–81	At discharge	Fair (any symptom of pain or cranial nerve involvement) or poor prognosis (cognitive impairment, disturbance of consciousness, multiple cranial nerve involvement, death)	Good 78%, fair 16%, poor 5% (only meningo-encephalitis patients), mortality not specified	Multivariate logistic regression: Worse outcome with >1.5 d to intravenous aciclovir
Mirouse et al. (72)	Retrospective, monocentre, France	*n* = 55	Median 53 (36–66)	In-hospital, after 1 year	mRS >2	25% mortality (in hospital), after 1 year 33% mortality, 22% significant disability (mRS 3–5), 36% favourable (mRS 0–2)	Multivariable analysis: Age, invasive mechanical ventilation

AE, autoimmune encephalitis; APACHE, acute physiology and chronic health evaluation score; CNS, central nervous system; CSF, cerebrospinal fluid; EEG, electroencephalography; FLAIR, fluid-attenuated inversion recovery; GOS, Glasgow outcome score; HSV, herpes simplex virus; ICU, intensive care unit; INFɣ, interferon gamma; IgG, immunoglobulin G; IQR, interquartile range; KPSS, Karnofsky performance status scale; MDRS, Mattis Dementia Rating Scale; MRI, magnetic resonance imaging; mRS, modified Rankin Scale; n.a., data not available; NMDAR, N-methyl-D-aspartate receptor; SAPS, simplified acute physiology score; VZV, varicella zoster virus. ^†^Given range or as indicated, *Despite citing the original description of the GOS in the publication, the score has been used in reverse order; for clarity we use the usual and initially described order of the score (73).

Mortality rates of HSV encephalitis reported in these studies were mostly between 5 and 20% (7, 11, 20, 22–24, 28, 30–38, 42, 46, 47, 49, 50, 52). Tan et al. described significantly increased mortality rates in immunocompromised compared to immunocompetent patients (36 versus 7%) (30). However, three studies reported no fatal cases (40, 53, 54), whereas five other studies found mortality rates of 24–64% (8, 27, 39, 41, 51). It is noteworthy that the studies reporting no mortality are most likely to have included more of the less severely affected patients. Kaewpoowat et al. (54) included 75% patients characterised as having meningitis and Kim et al. (40) reported a mean initial Glasgow Coma Scale (GCS) of 13.2 with altered mental status – a defining criterion for encephalitis – in only 25% of patients. Růžek et al. (53) did not provide further details on the clinical presentation of the study population and only divided the study population retrospectively into two groups: “mild” following successful uncomplicated therapy with good outcome or “severe” describing a severe course accompanied by acute neurological signs. On the other hand, large studies with mortality rates between 10 and 17% (47–49) included admission to the ICU as an inclusion criterion for study participation.

Outcome was reported to be favourable in 29–65% of survivors (8, 20, 22–24, 27, 29, 31–33, 37, 38, 40, 48, 49, 52, 53) and complete recovery was observed in 14–43% (7, 11, 22–24, 32, 33, 40, 51, 52). Interestingly, one prospective treatment study investigated an additional 3-month course of valaciclovir after standard aciclovir treatment. The authors described no or only mild residual neurocognitive deficits after 12 and 24 months in 86 and 90% of patients in the treatment and control group, respectively (34). A nationwide registry cohort study from Denmark noted a significantly increased risk of mortality (19% 1-year absolute excess mortality) in the first year and an increased risk of dementia in the first 5 years after detection of HSV in the CSF (50).

The factors most frequently associated with mortality and morbidity were age (8, 20, 22, 24, 28, 31, 37, 41, 42), pre-existing morbidity (7, 11, 42, 54), fever on admission (31, 47) and duration of fever after start of treatment (28), as well as lower GCS or a higher acute physiology and chronic health evaluation (APACHE) score on admission (8, 22, 24, 41). The following clinical parameters were also found to be associated with a worse outcome: longer interval between onset of main symptoms and hospitalisation (32), pre-existing immunocompromised state (30, 74) and status epilepticus, persistence of impaired consciousness, confusion or aphasia at day 5 of evolution (52), admission-to-MRI delay (52), need for mechanical ventilation (47) and length of stay in the ICU (52). Interestingly, direct admission to the ICU seems to be protective (47).

Development of autoimmune encephalitis (AE) within 3 months after HSV encephalitis has been described in up to 27% of cases (74% N-methyl-D-aspartate (NMDAR), 26% unknown antigens) (36, 43). Risk factors were younger age (≤4 years) and shorter interval between HSV encephalitis and detection of AE antibodies (43). Early detection of anti-NMDAR antibodies was associated with an overall increase in inflammatory CSF response and worse outcome (35, 36).

Regarding laboratory parameters, lower CSF cell count and initially negative HSV PCR were found to be associated with worse outcome in immunocompromised patients (30). Interestingly, another study noted that of 273 HSV encephalitis patients, 11 had negative HSV PCR in the first lumbar puncture performed 1 day after symptom onset (49). An initial negative HSV PCR was associated with worse outcome. In-hospital mortality was 27% and modified Rankin Scale (mRS) ≥4 at 3 months in 73% of PCR-negative patients compared to 14 and 33%, respectively in PCR-positive HSV encephalitis patients. This difference was only partially explained by delayed start of aciclovir treatment (49). Controversially, Mulatero et al. reported that 13% (11/76) of patients had negative HSV PCR in the first lumbar puncture performed – mean 1.8 ± 3 days (range 0–17 days) – after admission (52). These patients had less severe disease, but no difference was seen in the outcome (52). Overall, studies reported between 4 and 13% initial false negative HSV PCR results (41, 47, 49, 52).

Levels of neurofilament (NFL) in CSF (36) and serum albumin levels on admission (28) have been associated with outcome. No direct correlation between viral load in CSF and clinical outcome has been found (8, 23, 27, 53). Xanthochromia (haem degradation products in the CSF leading to a yellowish appearance) is a rare condition in HSV encephalitis (38) and is associated with poor outcome (8). Most likely, xanthochromia reflects advanced brain infection with tissue necrosis (34).

Several studies mention imaging findings (33), of these, nine studies described them in more detail or focused on MRI findings and their prognostic value (32, 39–41, 44, 47, 48, 52), as summarised in Table 2. Extent of brain involvement seen on MRI at admission, especially bilateral temporal lobe involvement, has been described as a factor associated with poor prognosis (32). The study by Sili et al. (32) has limitations due to missing information on time from hospital admission to MRI, lack of detailed description of abnormal MRI sequences and the high proportion of “suspected” HSV encephalitis (48% of the study population). However, it has been confirmed that fluid-attenuated inversion recovery (FLAIR) MRI signal abnormalities affecting more than three brain lobes, as well as the presence of diffusion-weighted MRI signal abnormalities in the left thalamus, were independently associated with poor outcome (47, 48). The multicentre studies by Jaquet et al. (47) and Sarton et al. (48), analysed large cohorts of patients with a well-described study population. Both these studies (47, 48) analysed the same cohort of HSV encephalitis patients requiring ICU treatment; however, Sarton et al. (48) included fewer patients in their analysis and focused exclusively on MRI and functional outcomes after HSV encephalitis. In these two studies, the MRI acquisition took place a median of 3 days after hospital admission and 1 day after ICU admission and it was abnormal in 99.3% of patients, with FLAIR hyperintensities as the most important finding (48). Singh et al. also showed that restricted diffusion on MRI was associated with poor outcome (mRS ≥3) in elderly people (median age 66 years) at hospital discharge as well as 6–12 months later (41). In contrast, other studies showed no association between MRI findings and outcome (40, 55). The studies by Kim et al. (40) and Jordan et al. (55) both included a retrospective analysis, of 25 and 15 patients, respectively. In the study by Kim et al. (40) there was a surprisingly low number of abnormal MRI (64% FLAIR and 59% diffusion-weighted imaging (DWI)) compared to larger studies (32, 48). However, the authors did not explicitly report the proportion of normal and abnormal MRI in HSV patients and the value of the study is therefore limited.

**Table 2:** Reports with MRI data from patients with HSV encephalitis.

	GCS (median and range) or level of consciousness on admission	Seizures/status epilepticus	Mechanical ventilation	Patients from study with MRI	Time from admission to MRI in Median^†^ days (IQR)	Abnormal MRI	FLAIR/T2 abnormalities	DWI	Bilateral	>3 lobes involved
Sili et al. (32)	Somnolence, stupor or coma in 35%	55%	n.a.	106/106*	n.a.	95%	n.a.	n.a.	20%	“Extensive” 17%
Kalita et al. (39)	<13 in 93% <8 in 60%	83%/48%	n.a.	40/40	n.a.	n.a.	n.a.	n.a.	78%	n.a.
Kim et al. (40)	Mean 13 (range 5–15)	55%	n.a.	25/29	Mean 2.8 (range 0–7)	n.a.	64%	59%	27%	
Jordan et al. (55)	Somnolence, sopor, coma in 73%	67%	20%	15/15	Within 48 h	87%	80%	90%	53%	
Singh et al. (41)	14 (10–15)	47%/13%	36%	40/45	2 (1–8.5)		95%	50%	42%	
Bewersdorf et al. (44)	n.a.	22%	n.a.	18/18	Mean 5.5 (range 1–16)	94%	94%	65%	41%	
Jaquet et al. (47)	9 (6–12)	36%/10%	62%	225/259	3 (1–9)	98%	62%			33%
Sarton et al. (48)	9 (6–12)	70%/46%	69%	138/138	3 (1–8)	99%	98%	48%	36%	38%
Mulatero et al. (52)	n.a.	21%/11%	34%	76/76	Mean 5.8 (±8.2)	n.a.	n.a.	n.a.	n.a.	n.a.

n.a, data not available; *of the 106 patients included; 55 had positive CSF HSV PCR, 25 had negative CSF HSV PCR and 26 were not tested for CSF HSV by PCR; ^†^Median, if not otherwise indicated.

Delay of aciclovir initiation significantly worsens clinical outcome (20–22, 30, 32, 37, 41). However, a dosage of aciclovir that is higher than the recommended standard dose of 10 mg per kg body weight every 8 h together with an additional course of oral valaciclovir therapy after aciclovir treatment did not improve outcome (29, 34). Very recently Mulatero et al. described an association between worse outcome, body weight and aciclovir dosage and suggested a weight-adjusted dose regimen, increasing the dose for patients with lower body weight (of <79 kg) up to 15 mg/kg body weight, especially for patients with a body weight below 57 kg (52). The question whether additional treatment with corticosteroids is beneficial for long-term outcome has yet to be answered (24, 45, 75). In one prospective randomized, double-blind, placebo-controlled treatment trial that had to be stopped prematurely due to slow recruitment, adjunctive steroid treatment did not affect mortality or neurological sequelae (45).

### 3.2. Varicella zoster virus encephalitis

As mentioned above, VZV causes a wide range of clinical manifestations of infection of the nervous system. Most frequently VZV infection or reactivation affects the PNS causing ganglionitis and dermatomal rash or facial nerve palsy (64). Less frequently, patients present with encephalitis, meningitis, cerebellitis, myelitis, or stroke/vasculopathy (59, 60, 64, 76, 77). Therefore, VZV infection can usually be discriminated clinically from HSV infection by the typical rash – if present – and the clinical presentation. However, no clinical sign or symptom can discriminate clinically between VZV and HSV encephalitis in the very early phase.

In this review, we focus on studies investigating outcome and prognosis after an encephalitic or meningitic course of VZV infection. Studies exclusively investigating outcome after PNS infection, herpes zoster or after vasculopathy are beyond the scope of this review. We included 22 studies (16 retrospective, 6 prospective) from 12 countries worldwide into the review, as summarised in Table 1. The outcome was generally assessed at discharge, some studies had follow-up periods of 1 to 6 months and/or after 1–3 years.

Encephalitis and meningitis due to VZV infection has a mortality rate of 0–15%, with fatal cases more likely during an encephalitic disease course (11, 34, 43, 53, 54, 57, 59, 62–64, 67–70). Only two studies reported mortality rates as high as 33% (72) and 36% (70). The first of these included only patients with severe encephalitis requiring intensive care (median GCS at admission 12, and mechanical ventilation in 84%) (72). In the second study, all the patients who died had a meningoencephalitic course plus stroke and/or myelitis (70).

A precise estimation of clinical outcome of survivors is difficult because of the varying definitions of outcome between the studies, different time points of evaluation (from discharge to follow-up after 3 years). Often outcome is reported combining various clinical manifestations of VZV infection of the CNS, sometimes even including PNS infection. However, the largest prospective study of VZV encephalitis, which included 92 patients, reported full recovery in 49% of patients after 3 months (68). Another study, prospectively investigating various infectious causes and outcomes of encephalitis described complete recovery in 41% of VZV encephalitis patients after 3 years (59). Interestingly, in another publication from the same group, which investigated the overall long-term outcome in patients from the same cohort study on infectious encephalitis, only 33% of VZV patients were found to have made a complete recovery after 3 years (7). A third study, with a retrospective design, worth mentioning here, showed a favourable 1-year outcome (i.e., mRS 0–2) in 36% of the whole study population and in 48% (20/41, excluding patients who died) of ICU survivors (72).

Most studies on VZV meningitis observe a good overall outcome in 70–100% of patients (54, 56, 60, 62, 63, 65, 66, 70, 71), although persisting neurological sequelae in 0 (60, 66) up to 82% (70) of patients have been described in some studies. In a small case–control study on 14 patients with VZV CNS infection (4 with meningitis, 6 with encephalitis and 4 with radiculitis or polyneuropathy) mild cognitive deficits were seen more frequently in a follow-up examination after 3–4 years than in a control group (60).

Prognostic factors for a severe acute disease course are controversial: whereas three studies found that higher VZV DNA load in the CSF was associated with disease severity (57, 58, 63), this was not confirmed in another study (53). In the acute phase, skin rash has been reported in 43–91% of patients (54, 56–58, 63, 65, 67, 68, 70). Only a few studies report herpes zoster in less than 60% of patients (56, 62, 70), occurring in 30–70% before (57, 58, 68), at or after onset of neurological signs and symptoms (57, 58). A shorter interval between appearance of herpes zoster and onset of neurological signs and symptoms has been described as a negative prognostic factor for death or sequelae (65). Older age (11, 67, 68, 70, 72) and pre-existing comorbidities (11, 54, 69, 70), as well as an encephalitic course of disease (54, 56, 68), need for mechanical ventilation (72) or signs of vasculitis (68) were associated with a worse outcome.

A large Danish cohort study analysing data from the national health registry showed that immunosuppressive state and comorbidities (Charlson Comorbidity Index >1) were a risk factor for detecting VZV DNA in the CSF (69). Mortality was increased in this VZV cohort, especially in the first year of observation and in patients with immunosuppressive or comorbid conditions (69). An increased risk of dementia and epilepsy, but not psychiatric disease, was found in the same cohort during the observation period of 12 years (69). Immunosuppression was a risk factor for more severe disease, but was not associated with worse outcome as found in three other studies (65, 70, 72).

MRI findings in patients with neurological VZV infections have been mixed, with pathological findings in 5% up to 70% of meningitis and encephalitis patients during the acute phase (54, 56, 61, 65, 67, 68, 70–72). To our knowledge, MRI findings have not so far been evaluated for their potential to serve as prognostic parameters, most likely due to incomplete data sets and mainly nonspecific MRI findings.

Interestingly, contrary to one prospective study (68) and two retrospective studies (67, 72), Yan et al. recently identified delayed time to aciclovir treatment as an independent risk factor for worse outcome (71). In the study by Le Bot et al., a higher dose of intravenous aciclovir (15 mg/kg every 8 h) was not found to be protective (67).

## 4. Discussion

Various studies have addressed outcome and prognostic factors in patients with HSV and VZV encephalitis. Since HSV encephalitis is the most common cause of viral encephalitis worldwide, with published case definitions (1), more studies with a reasonable number of study subjects and defined inclusion criteria are available than for VZV encephalitis or meningitis. Most studies had an observational, retrospective design and outcome was assessed mostly over a period of a few months up to 1 year and occasionally up to 3 years or more.

Mortality rates for HSV encephalitis varied significantly, from no mortality (40, 53, 54) to 65% mortality (41), while most studies reported mortality rates between 5 and 20% (7, 11, 22–24, 28, 30–38, 42, 47, 49, 50, 52). For encephalitis and meningitis due to VZV infection, slightly lower mortality rates of 0–15% have been reported (11, 34, 43, 53, 54, 57, 62, 63, 66–70). However, studies that have looked only at an encephalitic disease course found mortality rates of 33–36% (70, 72). Increased overall mortality has been observed within the first year after HSV or VZV encephalitis (50, 68). The outcome data were similarly varied: whereas some studies of HSV encephalitis describe a good (28, 32) or even excellent outcome for survivors (34, 35), other studies have reported high morbidity rates (47, 49). Patients with meningitis associated with VZV infection seems to have a good overall outcome in 70–100% of cases (54, 56, 60, 63, 65, 70, 71), whereas an encephalitic disease course is associated with high rates of long-term morbidity (70, 72). However, rates of complete recovery from HSV and VZV are comparable: around 14–43% for HSV (7, 11, 22–24, 32, 33, 40, 52) and 33–49% for VZV encephalitis (7, 68).

These large differences in mortality and morbidity rates are mainly attributable to the very different study designs and the widely varying definitions of inclusion criteria and studies are often difficult to compare. The lack of standardised inclusion criteria and outcome measures results in inclusion of more or less severely neurologically affected patients and some studies on VZV even combine patients with infection of the CNS and PNS. In addition, the outcome is defined very differently across the studies, which again makes it difficult to draw conclusions. Many studies used the mRS or the GOS; however, the cut-off for favourable and unfavourable outcome, as well as time-points of outcome evaluation were set inconsistently. Only a minority of studies investigated outcome in different functional neurological domains (i.e., neurocognitive, motor residuals, sleep–wake disorders etc.) and subjective impacts of neurological sequelae on daily life from the patient’s perspective have not been studied so far.

Risk or outcome scores are widely used in different medical fields (i.e., ABCD2-score for stroke risk after transient ischemic attack, Ranson’s criteria for pancreatitis mortality etc.). In our literature review, we found no prototype predictive score for estimating long-term clinical outcome for patients with viral encephalitis, comparable to the disability score for children after Japanese encephalitis (78). Most likely, this reflects the non-uniform definition of outcome measures (79).

Neuroimaging features may be essential tools not only to confirm the diagnosis and rule out alternative diagnoses but also to estimate outcome of disease. Whenever available, MRI is clearly preferable to CT for the diagnosis of encephalitis, given its sensitivity and specificity (80–82). The sensitivity of MRI in detecting acute, infectious encephalitis varies according to the causative agent: Overall, 95 to 100% of patients with HSV encephalitis show typical MRI abnormalities (28, 41, 48), therefore, alternative diagnoses should be considered if typical MRI findings are absent. From the largest MRI studies that focused on HSV encephalitis we can conclude that more extensive FLAIR lesions (>3 brain lobes affected) on the MRI acquired on admission are associated with higher mortality and morbidity (47, 48). On the other hand, VZV encephalitis may well be diagnosed despite normal MRI brain scans (55); this may explain why we found no study evaluating the prognostic value of MRI in VZV encephalitis.

To summarise and answer the main question posed in our review, many studies have been performed in patients with HSV and VZV encephalitis. HSV, more than VZV encephalitis, is associated with high mortality and long-term sequelae despite available therapy, and complete remission – at least for up to 3 years – is expected in fewer than half of patients. For further studies it is crucial to standardise inclusion criteria according to the case definitions (13) and use standardised outcome measures to allow comparability. Studies with longer follow-up periods and evaluation of functional impact of persisting sequelae on activities of daily life are also needed.

## Author contributions

LA, EH, AH, and AD participated in conception and organisation of review, literature search, and all stages of writing from initial draft to final product. All authors contributed to the article and approved the submitted version.

## Funding

AD has been personally funded by academic research grants from the Bangerter Rhyner Stiftung and University Bern. Open access funding was provided by University of Bern.

## Conflict of interest

The authors declare that the research was conducted in the absence of any commercial or financial relationships that could be construed as a potential conflict of interest.

## Publisher’s note

All claims expressed in this article are solely those of the authors and do not necessarily represent those of their affiliated organizations, or those of the publisher, the editors and the reviewers. Any product that may be evaluated in this article, or claim that may be made by its manufacturer, is not guaranteed or endorsed by the publisher.

## References

[1] Venkatesan A Tunkel AR Bloch KC Lauring AS Sejvar J Bitnun A. Case definitions, diagnostic algorithms, and priorities in encephalitis: consensus statement of the international encephalitis consortium. Clin Infect Dis. (2013) 57:1114–28. doi: 10.1093/cid/cit458, PMID: 23861361 PMC3783060

[2] Granerod J Tam CC Crowcroft NS Davies NWS Borchert M Thomas SL. Challenge of the unknown: a systematic review of acute encephalitis in non-outbreak situations. Neurology. (2010) 75:924–32. doi: 10.1212/WNL.0b013e3181f11d65, PMID: 20820004

[3] Boucher A Herrmann JLL Morand P Buzelé R Crabol Y Stahl JPP. Epidemiology of infectious encephalitis causes in 2016. Médecine Mal Infect. (2017) 47:221–35. doi: 10.1016/j.medmal.2017.02.003 28341533

[4] Jmor F Emsley HC Fischer M Solomon T Lewthwaite P. The incidence of acute encephalitis syndrome in Western industrialised and tropical countries. Virol J. (2008) 5:134. doi: 10.1186/1743-422X-5-134, PMID: 18973679 PMC2583971

[5] Solomon T Michael BD Smith PE Sanderson F Davies NWS Hart IJ. Management of suspected viral encephalitis in adults - Association of British Neurologists and British Infection Association National Guidelines. J Infect. (2012) 64:347–73. doi: 10.1016/j.jinf.2011.11.014, PMID: 22120595

[6] Granerod J Ambrose HE Davies NW Clewley JP Walsh AL Morgan D. Causes of encephalitis and differences in their clinical presentations in England: a multicentre, population-based prospective study. Lancet Infect Dis. (2010) 10:835–44. doi: 10.1016/S1473-3099(10)70222-X, PMID: 20952256

[7] Mailles A De Broucker T Costanzo P Martinez-Almoyna L Vaillant V Stahl JP. Long-term outcome of patients presenting with acute infectious encephalitis of various causes in France. Clin Infect Dis. (2012) 54:1455–64. doi: 10.1093/cid/cis226, PMID: 22460967

[8] Poissy J Champenois K Dewilde A Melliez H Georges H Senneville E. Impact of herpes simplex virus load and red blood cells in cerebrospinal fluid upon herpes simplex meningo-encephalitis outcome. BMC Infect Dis. (2012) 12:356. doi: 10.1186/1471-2334-12-356 23245564 PMC3560250

[9] Ungureanu A van der Meer J Bicvic A Abbuehl L Chiffi G Jaques L. Meningitis, meningoencephalitis and encephalitis in Bern: an observational study of 258 patients. BMC Neurol. (2021) 21:474. doi: 10.1186/s12883-021-02502-3, PMID: 34872509 PMC8647376

[10] Dagsdóttir HM Sigurðardóttir B Gottfreðsson M Kristjánsson M Löve A Baldvinsdóttir GE. Herpes simplex encephalitis in Iceland 1987–2011. Springerplus. (2014) 3:524. doi: 10.1186/2193-1801-3-524, PMID: 25279315 PMC4174550

[11] Mailles A Stahl JP. Infectious encephalitis in France in 2007: a national prospective study. Clin Infect Dis. (2009) 49:1838–47. doi: 10.1086/648419, PMID: 19929384

[12] Granerod J Cousens S Davies NWS Crowcroft NS Thomas SL. New estimates of incidence of encephalitis in England. Emerg Infect Dis. (2013) 19:1455–62. doi: 10.3201/eid1909.130064, PMID: 23969035 PMC3810913

[13] Tunkel AR Glaser CA Bloch KC Sejvar JJ Marra CM Roos KL. The Management of Encephalitis: clinical practice guidelines by the Infectious Diseases Society of America. Clin Infect Dis. (2008) 47:303–27. doi: 10.1086/589747, PMID: 18582201

[14] Whitley RJ. Herpes simplex encephalitis: adolescents and adults. Antivir Res. (2006) 71:141–8. doi: 10.1016/j.antiviral.2006.04.002 16675036

[15] Bradshaw MJ Venkatesan A. Herpes simplex virus-1 encephalitis in adults: pathophysiology, diagnosis, and management. Neurotherapeutics. (2016) 13:493–508. doi: 10.1007/s13311-016-0433-7, PMID: 27106239 PMC4965403

[16] Whitley RJ Alford CA Hirsch MS Schooley RT Luby JP Aoki FY. Vidarabine versus acyclovir therapy in herpes simplex encephalitis. N Engl J Med. (1986) 314:144–9. doi: 10.1056/NEJM198601163140303, PMID: 3001520

[17] Sköldenberg B Alestig K Burman L Forkman A Lövgren K Norrby R. Acyclovir versus vidarabine in herpes simplex encephalitis. Lancet. (1984) 2:707–11. doi: 10.1016/S0140-6736(84)92623-0 6148470

[18] Gershon AA Breuer J Cohen JI Cohrs RJ Gershon MD Gilden D. Varicella zoster virus infection. Nat Rev Dis Prim. (2015) 1:15016. doi: 10.1038/nrdp.2015.16, PMID: 27188665 PMC5381807

[19] Taba P Schmutzhard E Forsberg P Lutsar I Ljøstad U Mygland Å. EAN consensus review on prevention, diagnosis and management of tick-borne encephalitis. Eur J Neurol. (2017) 24:1214–e61. doi: 10.1111/ene.13356, PMID: 28762591

[20] McGrath N Anderson NE Croxson MC Powell KF. Herpes simplex encephalitis treated with acyclovir: diagnosis and long-term outcome. J Neurol Neurosurg Psychiatry. (1997) 63:321–6. doi: 10.1136/jnnp.63.3.321, PMID: 9328248 PMC2169720

[21] Utley TF Ogden JA Gibb A McGrath N Anderson NE. The long-term neuropsychological outcome of herpes simplex encephalitis in a series of unselected survivors. Neuropsychiatry Neuropsychol Behav Neurol. (1997) 10:180–9. http://www.ncbi.nlm.nih.gov/pubmed/9297711. PMID: 9297711

[22] Raschilas F Wolff M Delatour F Chaffaut C De Broucker T Chevret S. Outcome of and prognostic factors for herpes simplex encephalitis in adult patients: results of a multicenter study. Clin Infect Dis. (2002) 35:254–60. doi: 10.1086/341405, PMID: 12115090

[23] Kamei S Takasu T Morishima T Mizutani T. Serial changes of intrathecal viral loads evaluated by Chemiluminescence assay and nested PCR with Aciclovir treatment in herpes simplex virus encephalitis. Intern Med. (2004) 43:796–801. doi: 10.2169/internalmedicine.43.796, PMID: 15497513

[24] Kamei S. Evaluation of combination therapy using aciclovir and corticosteroid in adult patients with herpes simplex virus encephalitis. J Neurol Neurosurg Psychiatry. (2005) 76:1544–9. doi: 10.1136/jnnp.2004.049676 16227548 PMC1739396

[25] Kamei S Taira N Ishihara M Sekizawa T Morita A Miki K. Prognostic value of cerebrospinal fluid cytokine changes in herpes simplex virus encephalitis. Cytokine. (2009) 46:187–93. doi: 10.1016/j.cyto.2009.01.004, PMID: 19261488

[26] Taira N Kamei S Morita A Ishihara M Miki K Shiota H. Predictors of a prolonged clinical course in adult patients with herpes simplex virus encephalitis. Intern Med. (2009) 48:89–94. doi: 10.2169/internalmedicine.48.1445, PMID: 19145052

[27] Hjalmarsson A Granath F Forsgren M Brytting M Blomqvist P Sköldenberg B. Prognostic value of intrathecal antibody production and DNA viral load in cerebrospinal fluid of patients with herpes simplex encephalitis. J Neurol. (2009) 256:1243–51. doi: 10.1007/s00415-009-5106-6, PMID: 19353228

[28] Riera-Mestre A Gubieras L Martínez-Yelamos S Cabellos C Fernández-Viladrich P. Adult herpes simplex encephalitis: fifteen years’ experience. Enferm Infecc Microbiol Clin. (2009) 27:143–7. doi: 10.1016/j.eimc.2008.05.006, PMID: 19306713

[29] Stahl JP Mailles A De Broucker T. Herpes simplex encephalitis and management of acyclovir in encephalitis patients in France. Epidemiol Infect. (2012) 140:372–81. doi: 10.1017/S0950268811000483, PMID: 21470440

[30] Tan IL McArthur JC Venkatesan A Nath A. Atypical manifestations and poor outcome of herpes simplex encephalitis in the immunocompromised. Neurology. (2012) 79:2125–32. doi: 10.1212/WNL.0b013e3182752ceb, PMID: 23136265 PMC3511927

[31] Riancho J Delgado-Alvarado M Sedano MJ Polo JM Berciano J. Herpes simplex encephalitis: clinical presentation, neurological sequelae and new prognostic factors. Ten years of experience. Neurol Sci. (2013) 34:1879–81. doi: 10.1007/s10072-013-1475-9, PMID: 23780666

[32] Sili U Kaya A Mert A HSV Encephalitis Study Group. Herpes simplex virus encephalitis: clinical manifestations, diagnosis and outcome in 106 adult patients. J Clin Virol. (2014) 60:112–8. doi: 10.1016/j.jcv.2014.03.010 24768322

[33] Jouan Y Grammatico-Guillon L Espitalier F Cazals X François P Guillon A. Long-term outcome of severe herpes simplex encephalitis: a population-based observational study. Crit Care. (2015) 19:345. doi: 10.1186/s13054-015-1046-y, PMID: 26387515 PMC4576407

[34] Gnann JW Sköldenberg B Hart J Aurelius E Schliamser S Studahl M. Herpes simplex encephalitis: lack of clinical benefit of long-term valacyclovir therapy. Clin Infect Dis. (2015) 61:683–91. doi: 10.1093/cid/civ369, PMID: 25956891 PMC4542890

[35] Westman G Aurelius E Ahlm C Blennow K Eriksson K Lind L. Cerebrospinal fluid biomarkers of brain injury, inflammation and synaptic autoimmunity predict long-term neurocognitive outcome in herpes simplex encephalitis. Clin Microbiol Infect. (2021) 27:1131–6. doi: 10.1016/j.cmi.2020.09.031, PMID: 32979577

[36] Westman G Studahl M Ahlm C Eriksson BM Persson B Rönnelid J. N-methyl-D-aspartate receptor autoimmunity affects cognitive performance in herpes simplex encephalitis. Clin Microbiol Infect. (2016) 22:934–40. doi: 10.1016/j.cmi.2016.07.028, PMID: 27497810

[37] Erdem H Cag Y Ozturk-Engin D Defres S Kaya S Larsen L. Results of a multinational study suggest the need for rapid diagnosis and early antiviral treatment at the onset of herpetic meningoencephalitis. Antimicrob Agents Chemother. (2015) 59:3084–9. doi: 10.1128/AAC.05016-14, PMID: 25779579 PMC4432209

[38] Cag Y Erdem H Leib S Defres S Kaya S Larsen L. Managing atypical and typical herpetic central nervous system infections: results of a multinational study. Clin Microbiol Infect. (2016) 22:568.e9–568.e17. doi: 10.1016/j.cmi.2016.03.027, PMID: 27085724

[39] Kalita J Misra UK Mani VE Bhoi SK. Can we differentiate between herpes simplex encephalitis and Japanese encephalitis? J Neurol Sci. (2016) 366:110–5. doi: 10.1016/j.jns.2016.05.017, PMID: 27288787

[40] Kim YS Jung KH Lee ST Kang BS Yeom JS Moon J. Prognostic value of initial standard EEG and MRI in patients with herpes simplex encephalitis. J Clin Neurol. (2016) 12:224–9. doi: 10.3988/jcn.2016.12.2.224, PMID: 26833985 PMC4828570

[41] Singh TD Fugate JE Hocker S Wijdicks EFMM Aksamit AJ Rabinstein AA. Predictors of outcome in HSV encephalitis. J Neurol. (2016) 263:277–89. doi: 10.1007/s00415-015-7960-8, PMID: 26568560

[42] Jørgensen LK Dalgaard LS Østergaard LJ Nørgaard M Mogensen TH. Incidence and mortality of herpes simplex encephalitis in Denmark: a nationwide registry-based cohort study. J Infect. (2017) 74:42–9. doi: 10.1016/j.jinf.2016.09.004, PMID: 27717782

[43] Armangue T Spatola M Vlagea A Mattozzi S Cárceles-Cordon M Martinez-Heras E. Frequency, symptoms, risk factors, and outcomes of autoimmune encephalitis after herpes simplex encephalitis: a prospective observational study and retrospective analysis. Lancet Neurol. (2018) 17:760–72. doi: 10.1016/S1474-4422(18)30244-8, PMID: 30049614 PMC6128696

[44] Bewersdorf JP Koedel U Patzig M Dimitriadis K Paerschke G Pfister H-W. Challenges in HSV encephalitis: normocellular CSF, unremarkable CCT, and atypical MRI findings. Infection. (2019) 47:267–73. doi: 10.1007/s15010-018-1257-7, PMID: 30506479

[45] Meyding-Lamadé U Jacobi C Martinez-Torres F Lenhard T Kress B Kieser M. The German trial on Aciclovir and corticosteroids in herpes-simplex-virus-encephalitis (GACHE): a multicenter, randomized, double-blind, placebo-controlled trial. Neurol Res Pract. (2019) 1:26. doi: 10.1186/s42466-019-0031-3, PMID: 33324892 PMC7650106

[46] Oud L. Herpes simplex virus encephalitis: patterns of epidemiology and outcomes of patients admitted to the intensive care unit in Texas, 2008 - 2016. J Clin Med Res. (2019) 11:773–9. doi: 10.14740/jocmr4025, PMID: 31803321 PMC6879041

[47] Jaquet P de Montmollin E Dupuis C Sazio C Conrad M Susset V. Functional outcomes in adult patients with herpes simplex encephalitis admitted to the ICU: a multicenter cohort study. Intensive Care Med. (2019) 45:1103–11. doi: 10.1007/s00134-019-05684-0 31292686

[48] Sarton B Jaquet P Belkacemi D De Montmollin E Bonneville F Sazio C. Assessment of magnetic resonance imaging changes and functional outcomes among adults with severe herpes simplex encephalitis. JAMA Netw Open. (2021) 4:e2114328. doi: 10.1001/jamanetworkopen.2021.14328, PMID: 34313743 PMC8317014

[49] de Montmollin E Dupuis C Jaquet P Sarton B Sazio C Susset V. Herpes simplex virus encephalitis with initial negative polymerase chain reaction in the cerebrospinal fluid: prevalence, associated factors, and clinical impact. Crit Care Med. (2022) 50:e643–8. doi: 10.1097/CCM.0000000000005485 35167501

[50] Hansen A-BE Vestergaard H Dessau RB Bodilsen J Andersen NS Omland LH. Long-term survival, morbidity, social functioning and risk of disability in patients with a herpes simplex virus type 1 or type 2 central nervous system infection, Denmark, 2000–2016. Clin Epidemiol. (2020) 12:745–55. doi: 10.2147/CLEP.S256838 32765109 PMC7371560

[51] Müller-Jensen L Zierold S Versluis JM Boehmerle W Huehnchen P Endres M. Characteristics of immune checkpoint inhibitor-induced encephalitis and comparison with HSV-1 and anti-LGI1 encephalitis: a retrospective multicentre cohort study. Eur J Cancer. (2022) 175:224–35. doi: 10.1016/j.ejca.2022.08.009 36155116

[52] Mulatero M Boucekine M Felician O Boussen S Kaplanski G Rossi P. Herpetic encephalitis: which treatment for which body weight? J Neurol. (2022) 269:3625–35. doi: 10.1007/s00415-022-10981-8, PMID: 35099587

[53] Růžek D Piskunova N Žampachová E. High variability in viral load in cerebrospinal fluid from patients with herpes simplex and varicella-zoster infections of the central nervous system. Clin Microbiol Infect. (2007) 13:1217–9. doi: 10.1111/j.1469-0691.2007.01831.x, PMID: 17953699

[54] Kaewpoowat Q Salazar L Aguilera E Wootton SH Hasbun R. Herpes simplex and varicella zoster CNS infections: clinical presentations, treatments and outcomes. Infection. (2016) 44:337–45. doi: 10.1007/s15010-015-0867-6, PMID: 26680781

[55] Jordan B Kösling S Emmer A Koch A Müller T Kornhuber M. A study on viral CNS inflammation beyond herpes encephalitis. J Neurovirol. (2016) 22:763–73. doi: 10.1007/s13365-016-0452-5, PMID: 27173398

[56] Lee GH Kim J Kim HW Cho JW. Herpes simplex viruses (1 and 2) and varicella-zoster virus infections in an adult population with aseptic meningitis or encephalitis: a nine-year retrospective clinical study. Medicine (Baltimore). (2021) 100:e27856. doi: 10.1097/MD.0000000000027856, PMID: 34797322 PMC8601327

[57] Aberle SW Aberle JH Steininger C Puchhammer-Stöckl E. Quantitative real time PCR detection of varicella-zoster virus DNA in cerebrospinal fluid in patients with neurological disease. Med Microbiol Immunol. (2005) 194:7–12. doi: 10.1007/s00430-003-0202-1 14997388

[58] Persson A Bergström T Lindh M Namvar L Studahl M. Varicella-zoster virus CNS disease-Viral load, clinical manifestations and sequels. J Clin Virol. (2009) 46:249–53. doi: 10.1016/j.jcv.2009.07.014, PMID: 19709927

[59] De Broucker T Mailles A Chabrier S Morand P Stahl J-P. Acute varicella zoster encephalitis without evidence of primary vasculopathy in a case-series of 20 patients. Clin Microbiol Infect. (2012) 18:808–19. doi: 10.1111/j.1469-0691.2011.03705.x, PMID: 22085160

[60] Grahn A Nilsson S Nordlund A Lindén T Studahl M. Cognitive impairment 3 years after neurological varicella-zoster virus infection: a long-term case control study. J Neurol. (2013) 260:2761–9. doi: 10.1007/s00415-013-7057-1, PMID: 23900759

[61] Grahn A Hagberg L Nilsson S Blennow K Zetterberg H Studahl M. Cerebrospinal fluid biomarkers in patients with varicella-zoster virus CNS infections. J Neurol. (2013) 260:1813–21. doi: 10.1007/S00415-013-6883-5, PMID: 23471614

[62] Hong HL Lee EM Sung H Kang JK Lee SA Choi SH. Clinical features, outcomes, and cerebrospinal fluid findings in adult patients with central nervous system (CNS) infections caused by varicella-zoster virus: comparison with enterovirus CNS infections. J Med Virol. (2014) 86:2049–54. doi: 10.1002/JMV.23902, PMID: 24532558

[63] Rottenstreich A Oz ZK Oren I. Association between viral load of varicella zoster virus in cerebrospinal fluid and the clinical course of central nervous system infection. Diagn Microbiol Infect Dis. (2014) 79:174–7. doi: 10.1016/j.diagmicrobio.2014.02.015, PMID: 24666705

[64] Skripuletz T Pars K Schulte A Schwenkenbecher P Yildiz Ö Ganzenmueller T. Varicella zoster virus infections in neurological patients: a clinical study. BMC Infect Dis. (2018) 18:1–11. doi: 10.1186/s12879-018-3137-2, PMID: 29801466 PMC5970536

[65] Corral C Quereda C Muriel A Martínez-Ulloa PL González-Gómez FJ Corral Í. Clinical spectrum and prognosis of neurological complications of reactivated varicella-zoster infection: the role of immunosuppression. J Neurovirol. (2020) 26:696–703. doi: 10.1007/s13365-020-00872-x, PMID: 32696182

[66] Tabaja H Sharara SL Abi Aad Y Beydoun N Tabbal S Makki A. Varicella zoster virus infection of the central nervous system in a tertiary care center in Lebanon. Médecine Mal Infect. (2020) 50:280–7. doi: 10.1016/J.MEDMAL.2019.08.005, PMID: 31526545

[67] Le Bot A Ballerie A Pronier C Bénézit F Reizine F Tas M. Characteristics and outcome of varicella-zoster virus central nervous system infections in adults. Eur J Clin Microbiol Infect Dis. (2021) 40:2437–42. doi: 10.1007/s10096-021-04245-y 33907935

[68] Herlin LK Hansen KS Bodilsen J Larsen L Brandt C Andersen CØ. Varicella zoster virus encephalitis in Denmark from 2015 to 2019-a Nationwide prospective cohort study. Clin Infect Dis. (2021) 72:1192–9. doi: 10.1093/cid/ciaa185, PMID: 32103249

[69] Omland LH Vestergaard HT Dessau RB Bodilsen J Andersen NS Christiansen CB. Characteristics and long-term prognosis of Danish patients with varicella zoster virus detected in cerebrospinal fluid compared with the background population. J Infect Dis. (2021) 224:850–9. doi: 10.1093/infdis/jiab013, PMID: 33417703

[70] Lenfant T L’Honneur AS Ranque B Pilmis B Charlier C Zuber M. Neurological complications of varicella zoster virus reactivation: prognosis, diagnosis, and treatment of 72 patients with positive PCR in the cerebrospinal fluid. Brain Behav. (2022) 12:e2455. doi: 10.1002/brb3.2455, PMID: 35040287 PMC8865153

[71] Yan Y Yuan Y Wang J Zhang Y Liu H Zhang Z. Meningitis/meningoencephalitis caused by varicella zoster virus reactivation: a retrospective single-center case series study. Am J Transl Res. (2022) 14:491–500. 35173869 PMC8829630

[72] Mirouse A Sonneville R Razazi K Merceron S Argaud L Bigé N. Neurologic outcome of VZV encephalitis one year after ICU admission: a multicenter cohort study. Ann Intensive Care. (2022) 12:32. doi: 10.1186/s13613-022-01002-y, PMID: 35380296 PMC8982685

[73] Jennett B Bond M. Assessment of outcome after severe brain damage. A practical scale. Lancet. (1975) 305:480–4. doi: 10.1016/S0140-6736(75)92830-5 46957

[74] Mateen FJ Miller SA Aksamit AJ. Herpes simplex virus 2 encephalitis in adults. Mayo Clin Proc. (2014) 89:274–5. doi: 10.1016/j.mayocp.2013.12.003 24485138

[75] Whitfield T Fernandez C Davies K Defres S Griffiths M Hooper C. Protocol for DexEnceph: a randomised controlled trial of dexamethasone therapy in adults with herpes simplex virus encephalitis. BMJ Open. (2021) 11:e041808. doi: 10.1136/bmjopen-2020-041808, PMID: 34301646 PMC8728349

[76] Nagel MA Niemeyer CS Bubak AN. Central nervous system infections produced by varicella zoster virus. Curr Opin Infect Dis. (2020) 33:273–8. doi: 10.1097/QCO.0000000000000647 32332223 PMC13183292

[77] Koskiniemi M Piiparinen H Rantalaiho T Eränkö P Färkkilä M Räihä K. Acute central nervous system complications in varicella zoster virus infections. J Clin Virol. (2002) 25:293–301. doi: 10.1016/S1386-6532(02)00020-3 12423693

[78] Lewthwaite P Begum A Ooi MH Faragher B Lai BF Sandaradura I. Disability after encephalitis: development and validation of a new outcome score. Bull World Health Organ. (2010) 88:584–92. doi: 10.2471/BLT.09.071357, PMID: 20680123 PMC2908971

[79] Van Den TH Easton A Hooper C Mullin J Fish J Carson A. How should we define a ‘good’ outcome from encephalitis? A systematic review of the range of outcome measures used in the long-term follow-up of patients with encephalitis. Clin Med (Northfield Il). (2022) 22:145–8. doi: 10.7861/clinmed.2021-0505, PMID: 35197253 PMC8966817

[80] Steiner I Budka H Chaudhuri A Koskiniemi M Sainio K Salonen O. Viral meningoencephalitis: a review of diagnostic methods and guidelines for management. Eur J Neurol. (2010) 17:999–e57. doi: 10.1111/j.1468-1331.2010.02970.x 20236175

[81] Schroth G Kretzschmar K Gawehn J Voigt K. Advantage of magnetic resonance imaging in the diagnosis of cerebral infections. Neuroradiology. (1987) 29:120–6. doi: 10.1007/BF00327535 3587584

[82] Bertrand A Leclercq D Martinez-Almoyna L Girard N Stahl JP De-Broucker T. IRM des encéphalites aiguës infectieuses de l’adulte. Med Mal Infect. (2017) 47:195–205. doi: 10.1016/j.medmal.2017.01.002 28268128

