# Supplementary material for: Can we forecast poor outcome in herpes simplex and varicella zoster encephalitis? A narrative review
Source: Front Neurol. 2023 Jun 26;14:1130090. doi: 10.3389/fneur.2023.1130090 (PMC10331601; doi:10.3389/fneur.2023.1130090)

Supplementary figure 1: search and selection strategy

1. Pubmed Search Query: ("encephalitis, herpes simplex"[MeSH Terms] OR ("encephalitis"[All Fields] AND "herpes"[All Fields] AND "simplex"[All Fields]) OR "herpes simplex encephalitis"[All Fields] OR ("herpes"[All Fields] AND "simplex"[All Fields] AND "encephalitis"[All Fields])) AND ("outcome"[All Fields] OR "outcomes"[All Fields])
2. Pubmed Search Query: ("encephalitis, varicella zoster"[MeSH Terms] OR ("encephalitis"[All Fields] AND "varicella"[All Fields] AND "zoster"[All Fields]) OR "varicella zoster encephalitis"[All Fields] OR ("varicella"[All Fields] AND "zoster"[All Fields] AND "encephalitis"[All Fields])) AND ("outcome"[All Fields] OR "outcomes"[All Fields])


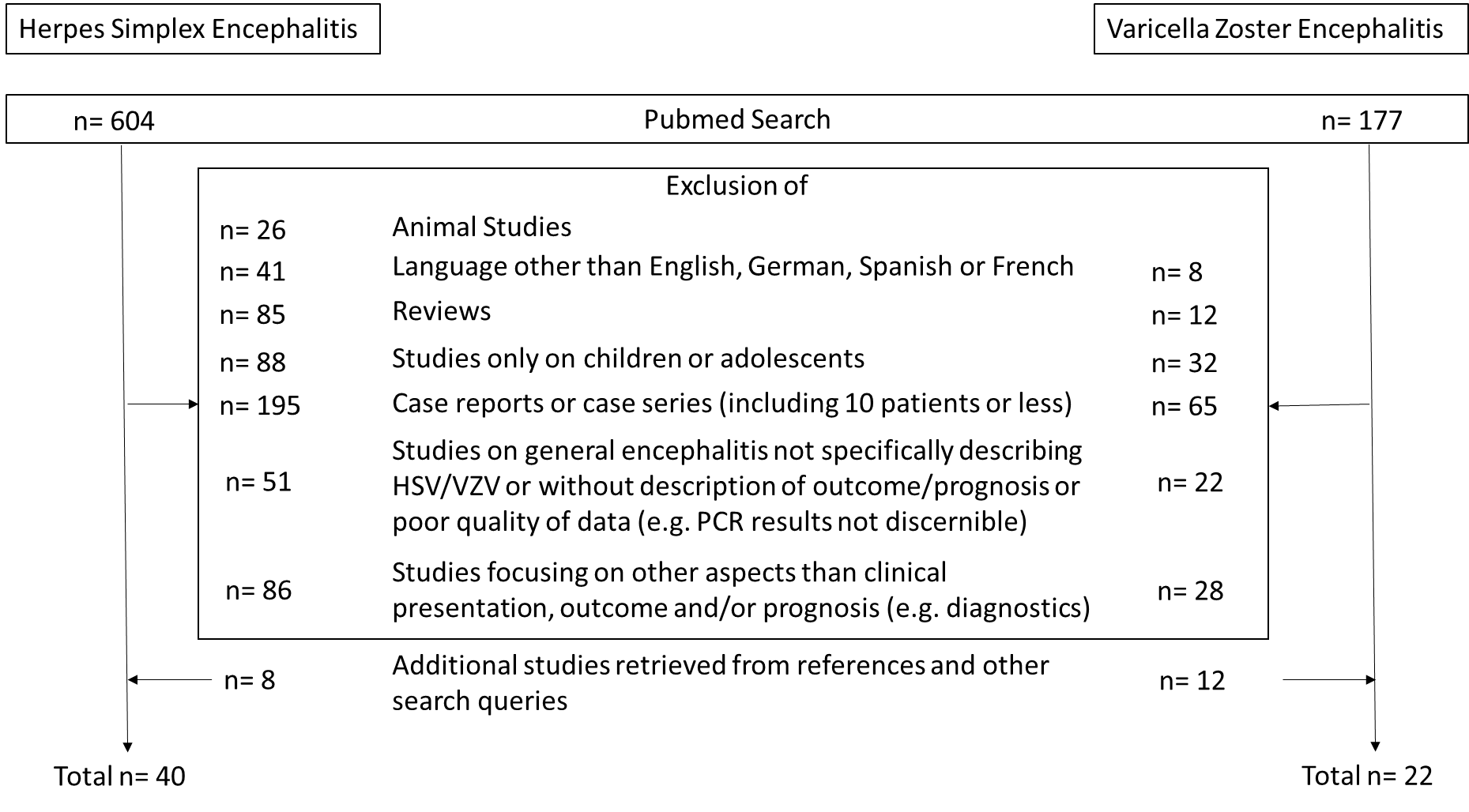

Supplement: Supplementary file 1 [file Table_1.DOCX]
